# Paternalistic Leadership and Job Embeddedness With Relation to Innovative Work Behaviors and Employee Job Performance: The Moderating Effect of Environmental Dynamism

**DOI:** 10.3389/fpsyg.2022.759088

**Published:** 2022-03-25

**Authors:** Mohammad Ali Yamin

**Affiliations:** Department of Human Resources Management, University of Jeddah, Jeddah, Saudi Arabia

**Keywords:** innovative work behavior, paternalistic leadership style, job embeddedness theory, environmental dynamism, structural equation modeling

## Abstract

The current study strives to examine the determinants of employee innovative work behavior and job performance. Therefore, an integrated research model is developed with the help of paternalistic leadership style and job embeddedness theory to investigate employee behavior toward innovative work behavior. The research model is extended with the moderating effect of environmental dynamism between the relationship of innovative work behavior and employee job performance. Data were collected from 411 employees working in small medium enterprises. For inferential analysis, the structural equation modeling (SEM) technique is used. Results of the structural equation modeling revealed that altogether paternalistic leadership style and factors underpinning job embeddedness theory have explained 52.1% of the variance in employee innovate work behavior. The findings of this research suggest that managers and policy makers should focus on benevolent leadership, moral leadership, and on-the-job embeddedness to boost employee job performance and innovative work behavior.

## Introduction

In today’s dynamic and complex environment, organizations need to incorporate innovative strategies to gain competitive advantages. It is noted that leadership style profoundly influences employee innovative work behavior (Shanker et al., 2017). Hou et al. (2019) postulated that leadership style had a significant impact on employee innovative work behavior. Therefore, examining the paternalistic leadership style toward employee innovative work behavior is the main point of discussion in this study. The paternalistic leadership style is different from conventional leadership and has been found in traditional Asian culture. The paternalistic leadership style works like a father role in a family who cares for his family, loves them, guides them, and supports them in each step of life (Hou et al., 2019). Authors like Erben and Güneşer (2008) asserted that implementing paternalistic leadership style in organizations increases employee commitment. Similarly, Chen et al. (2014) postulated that paternalistic leadership style boosts employee creativity and brings innovation to the workplace. Despite several useful outcomes, the impact of paternalistic leadership style in the context of employee innovative work behavior is less studied (Shanker et al., 2017; Hou et al., 2019). Authors like Khan and Mohiya (2020) asserted that little has been discussed about employee innovative behavior and its impact on small medium enterprises’ (SMEs) performance in Saudi Arabia. To fill this research gap, the current study investigates the core dimensions of paternalistic leadership style namely moral leadership, authoritarian leadership, and benevolent leadership, and examines how these factors influence employee innovative work behavior and job performance.

**FIGURE 1:**
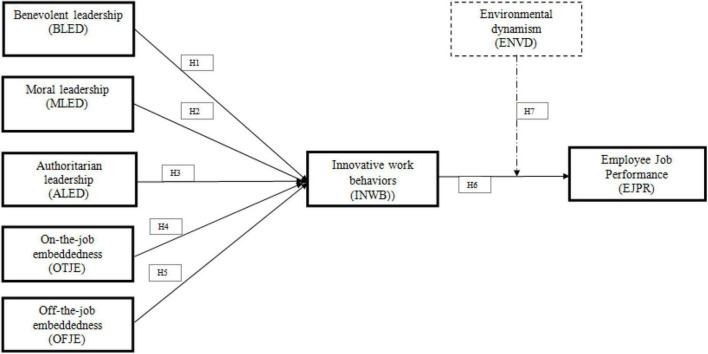
The proposed research framework.

Organizations are striving to understand which factors motivate employees to not leave an organization and this phenomenon is also known as job embeddedness in human resource literature. Job embeddedness theory incorporates two types of embeddedness, namely on-the-job embeddedness and off-the-job embeddedness (Holtom et al., 2006). On-the-job embeddedness explains that employees individually assess their job internally using cognitive factors that arise in the workplace. Therefore, off-the-job embeddedness indicates that employees evaluate their job through external factors such as community, social, and psychological embeddedness (Jiang et al., 2012). Studies have shown the significant impact of job embeddedness on employee innovative work behavior and job performance (Jiang et al., 2012; Ng and Feldman, 2010, 2013). Thus, the current study examines job embeddedness with relation to innovative work behavior. This study extends the body of knowledge with the moderating role of environmental dynamism. Authors like Yang and Li (2011) revealed that environmental dynamism creates uncertainty in business operations and negatively influences employee job performance. Following the above arguments and supported by Holtom et al. (2006); Shanker et al. (2017), and Hou et al. (2019), this study develops an integrated research model that comprises factors underpinning paternalistic leadership, employee job embeddedness, and environmental dynamism to investigate employee innovative work behavior and job performance. The research model is exhibited in Figure 1.

## Literature Review

### Paternalistic Leadership

The paternalistic leadership style comprises three factors namely benevolent leadership, moral leadership, and authoritarianism leadership. Benevolent leadership is a situation wherein leaders create an environment of employee wellbeing and provide continuous care both in the workplace and outside of the workplace (Dedahanov et al., 2016; Sahoo and Sahoo, 2019). In the workplace, a benevolent leader allows employees to make mistakes and provide opportunities to learn and correct these mistakes (Shao, 2019). Therefore, outside of the workplace benevolent leaders treat them like a family and motivate them to overcome life challenges (Pellegrini et al., 2010; Yamin and Mahasneh, 2018). These kinds of deeds make employees more energetic, enthusiastic, and committed toward innovative work behavior (Fu et al., 2013; Tian and Zhai, 2019; Norouzinik et al., 2021). Earlier studies have confirmed that paternalistic leadership significantly influences employee creativity, organizational commitment, and organizational performance (Erben and Güneşer, 2008; Chen et al., 2014; Zhang et al., 2015; Dedahanov et al., 2016). Thus, the following hypothesis is proposed:

H1: *Benevolent leadership has a positive influence on innovative work behavior.*

The moral leadership style is the second important element of paternalistic leadership. This kind of leader entertains employees with high spiritual virtues, shows unselfish and self-disciplined behavior toward employees, respects rules, and refuses to abuse power (Cheng et al., 2004; Islam et al., 2021). Authors like Gu et al. (2018) stated that a leader with moral characteristics influences employee behavior in such a way that they might imitate leader behavior and wish to become like moral leaders. Earlier studies showed the significant impact of moral leadership in predicting employee innovative work behavior (Cheng et al., 2004; Zhang et al., 2015; Gu et al., 2018; Kuenzi et al., 2021). Therefore, as backed up by earlier studies conducted by Cheng et al. (2004); Erben and Güneşer (2008), Pellegrini et al. (2010); Chen et al. (2014), Zhang et al. (2015), and Gu et al. (2018), the following hypothesis is proposed:

H2: *Moral leadership has a positive influence on innovative work behavior.*

The third dimension of paternalistic leadership is identified as authoritarian leadership which indicates leaders’ rigidness toward subordinates in the workplace. The authoritarian leader has strong autocratic control over employees and in return requires utter obedience from employees (Gu et al., 2018). The authoritarian kind of leadership style creates a depressing culture in organizations and diminishes employee creativity (Zhang et al., 2015; Cortellazzo et al., 2019; Player et al., 2019). Organizations practicing an authoritarian leadership style are less productive due to the fact that employees are afraid of being scolded by authoritative leaders (Pellegrini et al., 2010). Similarly, employees show reluctance toward creativity and innovation and prefer to use existing human resource practices. For organizational success, it is important to mitigate the negative aspects of authoritarian leadership style (Cheng et al., 2004; Zhang et al., 2015; Gu et al., 2018). Literature in paternalistic leadership confirmed that authoritarian leadership style had a negative impact on employee innovative behavior (Cheng et al., 2004; Pellegrini et al., 2010; Zhang et al., 2015; Gu et al., 2018). Thus, the following hypothesis is outlined:

H3: *Authoritarian leadership has a negative influence on innovative work behavior.*

### Job Embeddedness and Innovative Work Behavior

The concept of job embeddedness is extracted from voluntary employee turnover literature. In a human resource context, job embeddedness is seen as “*the degree in which [an] employee explains a set of characteristics that [enables them] to continue [in a] job instead of leaving an organization*” (Mitchell et al., 2001). Job embeddedness is further divided into two categories namely on-the-job embeddedness and off-the-job embeddedness. On-the-job embeddedness explains that employees individually assess their job internally using cognitive factors that arise in the workplace. According to Ng and Feldman (2013), employees with high on-the-job embeddedness have shown keenness in sharing new ideas. In addition to that, embedded employees spread innovation in an organization easily due to high on-the-job embeddedness (Ng and Feldman, 2010). On the other hand, off-the-job embeddedness indicates that employees evaluate their job through external factors such as community, social, and psychological embeddedness (Jiang et al., 2012; Morales-Sánchez and Pasamar, 2019). Employees with high off-the-job embeddedness have been shown to have a positive impact on employee job performance (Wheeler et al., 2012; Ng and Feldman, 2013). Earlier studies have shown that job embeddedness impacts employee innovative behavior (Feldman et al., 2012; Wheeler et al., 2012). Therefore, as backed up by existing work conducted by Mitchell et al. (2001); Jiang et al. (2012), Wheeler et al. (2012), and Ng and Feldman (2010, 2013), the following hypotheses are derived:

H4: *On-the-job embeddedness has a positive influence on innovative work behavior.*
H5: *Off-the-job embeddedness has a positive influence on innovative work behavior.*
H6: *Innovative work behavior has a positive influence on employee job performance.*

### Environmental Dynamism

Organization success is connected with the intensity of the external environment. Environmental dynamism is defined as “*the extent wherein organizations assess the rate of change and the degree of instability of the environment*” (Yang and Li, 2011). In an external dynamic environment, organizations’ operation and performance might be affected due to shifting customer choices, advancement in technologies, and fluctuation in demand and supply (Jansen et al., 2009; Murcio and Scalzo, 2021). In other words, environmental dynamism creates great uncertainty in business operations and negatively influences employee job performance (Wang et al., 2013). Literature indicates that innovation may be risker in an organization that is facing environmental changes (Wang et al., 2013). Therefore, understanding the relationship between environmental dynamism and employee innovative work behavior is important for employee job performance (Jansen et al., 2009; Yang and Li, 2011). Earlier studies confirmed that changes in the environment had a negative impact on employee innovative work behavior and job performance (Jansen et al., 2009; Yang and Li, 2011). Therefore, the current study extends the body of knowledge in this context and outlines the moderating role of environmental dynamism between innovative work behavior and employee job performance which is in line with Wang et al. (2013). Therefore, the following hypothesis is proposed:

H7: *Environmental dynamism negatively moderates the relationship between innovative work behavior and employee job performance.*

## Methodology

### Respondents and Data Collection

For the research design, this study has adopted a positivist paradigm. The followers of positivist paradigm believe in fresh data collection to investigate the phenomenon. The population of this study includes employees working in small medium organizations in Saudi Arabia. For the sample size calculation, the researcher used prior-power analysis consistent with earlier studies (Rahi, 2017a; Rahi et al., 2020). Results indicate that 300 responses were enough to conduct factor analysis. A set of 569 questionnaires was distributed among employees using a convenience sampling approach. According to Rahi et al. (2019a), the convenience sampling approach is appropriate when the list of the respondents is not at hand. In addition to that, the convenience sampling approach helps researchers to engage with actual respondents (Rahi et al., 2019b). Out of 569 distributed questionnaires, 456 were returned with a response rate of 80%. Of these responses 45 were discarded due to inappropriate filling in, in line with Rahi and Abd. Ghani (2019b). Thus, a total of 411 valid responses were used for structural equation modeling (SEM).

### Questionnaire Development

The theoretical model of this study is based on employee innovative work behavior and job performance with paternalistic leadership and job embeddedness theory. Therefore, a research questionnaire was developed comprising construct items. Construct items were adopted from previous literature and then adapted into the current research context. Construct items for innovative work behavior were adapted from De Jong and Den Hartog (2010). Employee job performance items were adapted from Janssen and Van Yperen (2004) and Khoreva and Wechtler (2018). Item scales for on-the-job embeddedness were adapted from Coetzer et al. (2018). Next to this, off-the-job embeddedness items were adopted from Felps et al. (2009) and then adapted into the innovative work behavior context. Construct items for environmental dynamism were adopted from Jaworski and Kohli (1993) and then adapted. The paternalistic leadership constructs which included authoritarian leadership, moral leadership, and benevolent leadership were adopted from Erben and Güneşer (2008), and then adapted into the current research context. All construct items were measured with a Likert point scale ranging from 1 for strongly disagree to 7 for strongly agree.

### Testing Common Method Variance Issue

For the research design, this study is quantitative in nature and investigates the phenomenon using fresh data. According to Samar and Mazuri (2019a), a study that collects data using a single source may be affected by common method variance bias. Therefore, testing common method variance bias before the structural equation model is important (Bader and Mohammad, 2019; Samar and Mazuri, 2019b; Yamin and Sweiss, 2020). In this study, the common method bias was tested with Harman’s single factor analysis which suggests that the variance explained by a single factor should not be greater than 40% (Podsakoff et al., 2003; Rahi and Abd. Ghani, 2019a). Results revealed that the variance explained by a single factor was only 14% which showed that common method variance (CMV) was not likely an issue in this research.

## Data Analysis and Results

The research model was tested with the latest statistical approach namely structural equation modeling (Hair et al., 2016). For structural equation modeling, the researcher used a two-step approach which included a measurement model and a structural model (Rahi, 2017b). The measurement model assesses construct reliability and validity, therefore the structural model tests the causal relationship between exogenous and endogenous variables. The details of the measurement model and structural model are given in the following sections.

### Measurement Model

The measurement model confirms convergent validity and discriminant validity of the constructs (Yamin, 2019). The convergent validity of the construct is achieved with Cronbach’s alpha, composite reliability, average variance extracted, and factor loadings of the constructs (Yamin, 2020a). In order to achieve construct reliability, the values of Cronbach’s alpha and composite reliability should be greater than 0.70, which indicates satisfactory reliability of the construct. Construct reliability is also assessed with average variance extract (AVE) following the criterion that the values of AVE should be higher than 0.50, meaning the construct is reliable. Finally, factor loading of the construct is tested following the criterion that loading should be greater than 0.60 as suggested by Rahi and Abd. Ghani (2019c). Results indicated that all the values of composite reliability, Cronbach’s alpha, average variance extracted, and factor loadings were adequate, hence confirming reliability of the constructs. Table 1 shows the results of the measurement model.

**TABLE 1:** Measurement model.

Questionnaire items	Loading	(α)	CR	Average variance extract (AVE)
ALED1: In my organization managers scold us when we fail to accomplish our daily work.	0.831	0.685	0.862	0.757
ALED2: In my organization we are bound to follow our manager’s rules otherness he/she will punish us severely.	0.908			
BLED1: Regardless of work relations, my organization managers care about my daily life.	0.764	0.816	0.878	0.643
BLED2: Managers in my organization express concern for my family members as well.	0.821			
BLED3: Managers in my organization guide me when I encounter problems.	0.795			
BLED4: Managers in my organization are like my family member whenever they meet with me.	0.826			
EJPR1: In my organization employees meet all the formal performance requirements of the job.	0.881	0.851	0.910	0.770
EJPR2: In my organization employees complete their duties as per the job description.	0.889			
EJPR3: In my organization employees never neglect aspects of their job in which they are bound to perform.	0.863			
ENVD1: The technological changes in our industry are rapid and unpredictable.	0.888	0.818	0.891	0.731
ENVD2: The environmental changes in our industry are intense and unpredictable.	0.857			
ENVD3: The action of local and foreign competitors in our market is changing rapidly.	0.819			
INWB1: In my organization employee suggestions improve products or services.	0.844	0.876	0.915	0.729
INWB2: In my organization employees actively participate in new product development or services.	0.862			
INWB3: Innovative work behavior suggests acquiring new knowledge externally in order to improve their job performance.	0.867			
INWB4: Innovative work behavior may increase by supporting people’s innovative ideas.	0.841			
MLED1: In my organization managers treat employees according to their qualities and envy others’ abilities and qualities.	0.833	0.866	0.909	0.713
MLED2: In my organization the manager does not take advantage of other employee’s virtues for personal gain.	0.846			
MLED3: In my organization the manager does not take credit of other employee’s achievements and contribution for personal gains.	0.874			
MLED4: In my organization the manager does not use back-door practices or personal relationships to get personal gains.	0.824			
OFJE1: The place where I live offers wonderful leisure activities including cultural and outdoor activities.	0.767	0.767	0.850	0.587
OFJE2: Off the job, I am engaged with community organizations such as mosques, churches, schools, and sport teams.	0.747			
OFJE3: Off the job, I am active in recreational and cultural activities in my area.	0.795			
OFJE4: I will miss my neighborhood if I leave my area where I live.	0.755			
OTJE1: On the job, I feel that I am a good match for my organization.	0.719	0.667	0.797	0.512
OTJE2: I will achieve most of my tasks if I continue my job with my organization.	0.395			
OTJE3: I will keep interacting with my peers if I stay with my organization.	0.870			
OTJE4: The idea for staying with this organization is excellent.	0.787			

The convergent validity of the research model was achieved with factor loadings, average variance extracted, Cronbach’s alpha, and composite reliability. Therefore, the discriminant validity of the research constructs was examined with Fornell and Larcker criterion (Fornell and Larcker, 1981). According to Rahi and Abd. Ghani (2018), discriminant validity confirms that the construct is dissimilar and measures different concepts. In order to achieve discriminant validity, the criterion is that the values of AVE should be greater than the correlation of the corresponding construct indicating that the construct is discriminant (Fornell and Larcker, 1981; Yamin and Sweiss, 2020; Yamin, 2020b). Table 2 depicts the results of discriminant validity herein the values of average variance extracted were greater than the correlation of the corresponding constructs. These findings confirmed the discriminant validity of the constructs.

**TABLE 2:** Discriminant validity.

Constructs	ALED	BLED	EJPR	ENVD	INWB	MLED	OFJE	OTJE
ALED	*0.870*							
BLED	0.265	*0.802*						
EJPR	0.214	0.540	*0.878*					
ENVD	0.031	0.152	0.220	*0.855*				
INWB	0.238	0.654	0.719	0.201	*0.854*			
MLED	0.333	0.295	0.496	0.334	0.455	*0.844*		
OFJE	0.545	0.329	0.269	0.027	0.332	0.251	*0.766*	
OTJE	0.366	0.398	0.341	0.111	0.397	0.244	0.398	*0.716*

The discriminant validity was tested by applying the heterotrait-monotrait ratio (HTMT) method (Rahi et al., 2018; Yamin and Alyoubi, 2020). This method suggests that the values of HTMT analysis should not be greater than 0.85 indicating adequate discriminant validity of the constructs (Gold and Arvind Malhotra, 2001; Kline, 2011; Samar et al., 2017; Yamin and Mahasneh, 2018). The results of HTMT analysis indicated that all HTMT values were less than 0.85 confirming that the construct was discriminant and measured distinct concepts. The results of HTMT analysis can be seen in Table 3.

**TABLE 3:** Discriminant validity with the heterotrait-monotrait ratio (HTMT) method.

Constructs	ALED	BLED	EJPR	ENVD	INWB	MLED	OFJE	OTJE
ALED								
BLED	0.348							
EJPR	0.277	0.640						
ENVD	0.052	0.188	0.258					
INWB	0.301	0.757	0.821	0.234				
MLED	0.432	0.347	0.576	0.393	0.514			
OFJE	0.751	0.408	0.325	0.071	0.400	0.302		
OTJE	0.514	0.519	0.430	0.163	0.493	0.304	0.522	

### Structural Model

The structural model assessment included calculation of the coefficient of determination *R*^2^, lateral multicollinearity, path coefficients, *t*-statistics, and significance level of the hypothesis. According to Hair et al. (2016), the measurement model confirms vertical multicollinearity, therefore it is important to check lateral multicollinearity of the construct using variance inflation factor (VIF) analysis. In order to achieve lateral collinearity, the criterion is that the values of the VIF should not be higher than 3.3 (Hair et al., 2016; Mohammad Ali, 2018). Results of the structural model revealed that the values of variance inflation factor were less than 3.3 when comparing endogenous variable VIF values with exogenous variables. These findings confirmed that lateral multicollinearity was not likely an issue in this study and in line with Yamin and Sweiss (2020). The results of the VIF analysis can be seen in Table 4.

**TABLE 4:** Assessing multicollinearity using variance inflation factor (VIF) statistics.

Constructs	Employee job performance	Innovative work behavior
Authoritarian leadership		1.552
Benevolent leadership		1.288
Environmental dynamism	1.042	
Innovative work behaviors	1.042	
Moral leadership		1.193
Off-the-job embeddedness		1.560
On-the-job embeddedness		1.357

#### Hypothesis Testing

The causal relationship between hypotheses was tested with beta value (β), significance level of the relationship, and *t*-statistics values. In addition to that, a bootstrapping procedure was used with re-samples of data (5,000) to mitigate the data normality issue (Sweiss and Yamin, 2020). The results of the hypotheses testing can be seen in Table 5.

**TABLE 5:** Hypothese.

Hypotheses	Relationship	Path–β	Standard deviation	*T*-statistics	*P*-values
H1	BLED → INWB	0.517	0.037	13.854	0.000
H2	MLED → INWB	0.278	0.042	6.583	0.000
H3	ALED → INWB	−0.085	0.047	1.794	0.037
H4	OTJE → INWB	0.117	0.045	2.579	0.005
H5	OFJE → INWB	0.092	0.049	1.897	0.029
H6	INWB → EJPR	0.703	0.031	22.601	0.000

Findings of the structural equation model indicated that benevolent leadership had a significant influence on innovative work behavior and statistically confirmed H1 (β = 0.517, *t*-value 13.854, significance *p* < 0.001). Moral leadership had a significant influence on employee innovative work behavior and supported H2 (β = 0.278, *t*-value 6.583, significance *p* < 0.001). Furthermore, the negative relationship between authoritarian leadership and innovative work behavior was found to be significant and supported H3 (β = −0.085, *t*-value 1.794, significance *p* < 0.05). Concerning job embeddedness theory, the results revealed that both constructs for on-the-job embeddedness and off-the-job embeddedness had a significant influence on innovative work behavior, hence confirming H4 and H5 (β = 0.117, *t*-value 2.579, significance *p* < 0.01; β = 0.092, *t*-value 1.897, significance *p* < 0.05). Innovative work behavior had a significant influence on employee job performance and statistically confirmed H6 (β = 0.703, *t*-value 22.601, significance *p* < 0.001). Finally, the structural model revealed the coefficient of determination *R*^2^. Results indicated that job embeddedness theory and paternalistic leadership explained an *R*^2^ of 52.1% of variance in employee innovative work behavior. Therefore, employee job performance was predicted by innovative work behavior and showed an *R*^2^ of 53.6% of variance in employee job performance. These findings indicated that the research model had substantial power to predict employee innovative work behavior and job performance. The path coefficient and significance level of the hypotheses can be seen in Appendix 1.

#### Effect Sizes (*f*^2^) and Predictive Relevance (*Q*^2^)

The structural model assessment revealed combined (*R*^2^) variance in innovative work behavior and employee job performance. Therefore, the coefficient of determination does not compute the single construct effect size (Rahi, 2018; Yamin, 2020c). Thus, the effect size of a single construct was estimated with effect size *f*^2^ analysis. Results indicated that benevolent leadership had a substantial effect size in predicting employee innovative work behavior. Therefore, all other constructs including on-the-job and off-the-job embeddedness, moral leadership, and authoritarian leadership had small effect sizes. Concerning employee job performance, results showed that innovative work behavior had a substantial effect size. Therefore, environmental dynamism had a small effect size in shaping employee job performance. The predictive relevance of the model was also tested to confirm whether the model had enough predictive power. The predictive power of the research model was tested with blindfolding procedure *Q*^2^ (Rahi, 2017a). Findings of the blindfolding procedure revealed that altogether authoritarian leadership, benevolent leadership, moral leadership, and on-the-job and off-the-job embeddedness had substantial predictive power *Q*^2^(36.7%) to predict employee innovative work behavior. Similarly, employee job performance was predicted by environmental dynamism and innovative work behavior and had substantial predictive power *Q*^2^(4.4%) to predict employee job performance. These findings confirmed that the integration of job embeddedness theory with leadership was valid and had an influential impact on employee innovative work behavior and employee job performance. The results of effect size *f*^2^ analysis and predictive relevance *Q*^2^ are exhibited in Table 6.

**TABLE 6:** Effect size analysis *f*^2^ and predictive relevance *Q*^2^.

Constructs	R^2^	Q^2^	(f^2^)	Decision
**Innovative work behavior**				
Innovative work behavior	0.521	0.367		
Authoritarian leadership			0.010	Small
Benevolent leadership			0.434	Substantial
Moral leadership			0.136	Small
On-the-job embeddedness			0.011	Small
Off-the-job embeddedness			0.021	Small
**Employee job performance**				
Employee job performance	0.536	0.404		
Environmental dynamism			0.013	Small
Innovative work behaviour			1.022	Substantial

*Effect size f^2^ indicates: 0.02, small; 0.15, medium; 0.35, substantial.*

### Importance Performance Matrix Analysis

A *post-hoc* analysis namely importance performance matrix analysis was employed to determine the importance and performance of the constructs. According to Hair et al. (2016), importance performance matrix analysis adds an additional dimension in structural model estimation and reveals construct importance and performance for managerial implication. The importance performance matrix analysis rescales the data from 0 to 100 and produces two output total effect and index values of the constructs (Hair et al., 2016). Findings of the importance performance matrix analysis showed that innovative work behavior had the highest importance values. Therefore, benevolent leadership exhibited the second highest importance values to predict employee job performance. Constructs like moral leadership and on-the-job embeddedness had an intermediate level of importance. Therefore, the importance of off-the-job embeddedness, environmental dynamism, and authoritarian leadership lagged behind benevolent leadership. Table 7 depicts the values of importance performance matrix analysis with importance and performance scores.

**TABLE 7:** The importance performance matrix analysis.

Latent constructs	Importance of employee job performance	Performance of employee job performance
Authoritarian leadership	–0.065	71.888
Benevolent leadership	0.420	67.951
Environmental dynamism	0.097	63.306
Innovative work behavior	0.727	66.903
Moral leadership	0.203	73.271
Off-the-job leadership	0.082	71.114
On-the-job leadership	0.105	65.805

**FIGURE 2:**
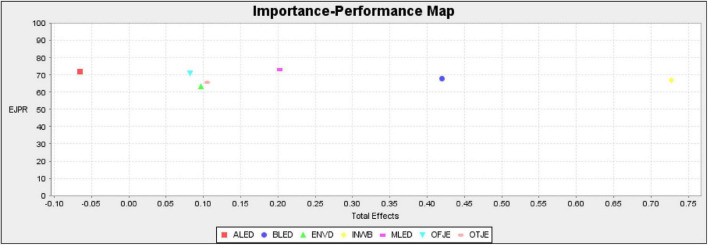
Importance performance matrix map.

Importance performance matrix analysis values can be seen in the importance performance matrix (IPMA) map as given in Figure 2. The IPMA map clearly shows that authoritarian leadership had the lowest importance values to predict employee job performance. Therefore, constructs like benevolent leadership, moral leadership, innovative work behavior, and on-the-job embeddedness were the most influential factors to predict employee job performance. For practical implication, it is recommended that human resource managers and policy makers should focus on benevolent leadership, moral leadership style, employee innovative work behavior, and on-the-job embeddedness in order to boost employee job performance.

### Moderating Analysis

**FIGURE 3:**
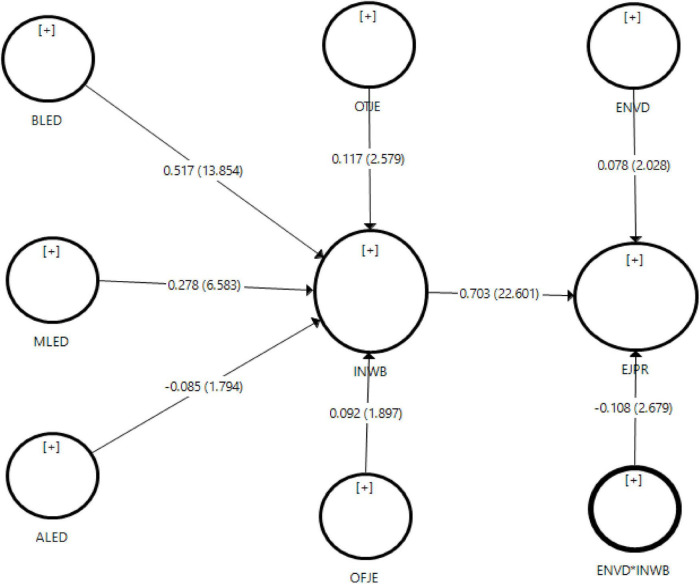
Moderating effect of environmental dynamism.

This study extends the body of knowledge by adding the moderating role of environmental dynamism between employee innovative work behavior and employee job performance. According to Wang et al. (2013), environmental dynamism creates great uncertainty in business operations and negatively influences employee job performance. Similarly, literature revealed that innovation in work may be risker in an organization that is facing environmental changes (Jansen et al., 2009; Yang and Li, 2011; Wang et al., 2013). Thus, the researcher hypothesized that *environmental dynamism negatively moderates the relationship between innovative work behavior and employee job performance.* For moderating analysis, the researcher used the product indicator approach as suggested by Hair et al. (2016). Before structural model estimation, the researcher computed the interaction effect of innovative work behavior and environmental dynamism. Results indicated that the environmental dynamism negatively moderated the relationship between employee innovative work behavior and employee job performance and supported H7 (β = −0.108, *t*- 2.679 < 0.05). Figure 3 shows the interaction effect of employee innovative work behavior and environmental dynamism with *t*-statistics.

## Discussion

Increase in globalization and rapid changes in the environment have required organizations to use innovative strategies in order to survive in this fierce and competitive business world. Therefore, understanding what factors influence employee innovative work behavior is important for organization success. In this essence, a research model was developed that combines leadership and job embeddedness factors. Findings of structural equation modeling revealed that benevolent leadership significantly influenced employee innovative work behavior, which is consistent with earlier studies by Fu et al. (2013) and Tian and Zhai (2019). Moral leadership had a significant influence on employee innovative work behavior, in line with Cheng et al. (2004); Zhang et al. (2015), and Gu et al. (2018). Aside from the positive relationship, the negative relationship between authoritarian leadership and innovative work behavior was significant and in line with previous research by Gu et al. (2018). Next to this, constructs underpinning job embeddedness theory demonstrated that on-the-job embeddedness and off-the-job embeddedness had a significant influence on innovative work behavior in line with Wheeler et al. (2012) and Ng and Feldman (2013). Similarly, innovative work behavior significantly influenced employee job performance, which is consistent with Ng and Feldman (2010). These findings confirmed that paternalistic leadership style and job embeddedness theory have a significant impact on employee innovative work behavior and job performance.

Concerning environmental dynamism, results confirmed that environmental dynamism negatively moderated the relationship between innovative work behavior and employee job performance, in line with a previous study by Wang et al. (2013). Effect size analysis indicated that benevolent leadership had a substantial effect size in predicting employee innovative work behavior. Therefore, all other constructs including on-the-job and off-the-job embeddedness, moral leadership, and authoritarian leadership had small effect sizes. In the extended research model, results showed that innovative work behavior had a substantial effect size when determining employee job performance with environmental dynamism and innovative work behavior. The predictive power of the model was tested with a blindfolding procedure. Findings revealed that the research model had substantial predictive power *Q*^2^(36.7%) to predict employee innovative work behavior. Similarly, innovative work behavior and environmental dynamism revealed substantial predictive power *Q*^2^(4.4%) to predict employee job performance. Finally, a substantial coefficient of determination was found with an *R*^2^ of 52.1% of variance in employee innovative work behavior and an *R*^2^ of 53.6% of variance in employee job performance. Therefore, the research model had a significant influence on predicting employee innovative work behavior and employee job performance.

### Theoretical Implications

The current research has added several contributions to theory and innovation literature. First, this study investigated employee innovative work behavior with paternalistic leadership style. Therefore, developing the relationship between paternalistic leadership style and innovative work behavior contributes to leadership literature in the context of innovative work behavior. Second, the current research has outlined job embeddedness theory to investigate employee innovative work behavior. Hence, examining the impact of job embeddedness characteristics toward innovative work behavior enhances the generalizability of job embeddedness theory. Third, the research model was extended with the moderating effect of environmental dynamism between innovative work behavior and employee job performance. Findings confirmed that the moderating effect of environmental dynamism between innovative work behavior and employee job performance had a negative impact and hence this manuscript contributes to organizational environmental-based literature. Finally, the development of the integrated research model that combined paternalistic leadership style factors and job embeddedness provides a clear direction to academic researchers that employee innovative work behavior could be established with paternalistic leadership and job embeddedness.

### Practical Implications

Practically, this study provides guidelines to human resource professionals while suggesting that highly embedded employees are more likely to engage in innovative work behavior compared to those who are less job-embedded. Similarly, a benevolent leadership style had the highest impact in determining employee innovative work behavior which in turn enhanced employee job performance. Similarly, the importance performance matrix analysis suggests that that human resource managers and policy makers should focus on benevolent leadership, moral leadership style, employee innovative work behavior, and job embeddedness factors in order to boost employee job performance. Another aspect of this study is to assess the environmental context in an organization. This study confirmed that environmental dynamism negatively moderates the relationship between innovative work behavior and employee job performance. Therefore, results indicate that organizations facing environmental dynamic issues should be careful in introducing innovative ideas into market. Precisely, this research suggests that managers and policy makers should focus on factors such as employee job embeddedness and paternalistic leadership style in order to increase employee innovative work behavior and employee job performance.

## Conclusion

The innovative work behavior of employees plays a key role in achieving the strategic goal of a firm. Therefore, investigating factors which impact employee innovative work behavior and employee job performance is important and should be taken into managerial consideration. The current study strived to examine the innovative work behavior of employees with job embeddedness and a paternalistic leadership style. The research model confirmed that factors underpinning job embeddedness and paternalistic leadership directly influence employee innovative work behaviors. The results of structural equation modeling revealed that job embeddedness theory and paternalistic leadership explained an *R*^2^ of 52.1% of variance in employee innovative work behavior. Therefore, employee job performance was predicted by innovative work behavior and showed an *R*^2^ of 53.6% of variance in employee job performance. Similarly, the effect size *f*^2^ analysis indicated that benevolent leadership had a substantial effect size in predicting employee innovative work behavior. Therefore, all other constructs including on-the-job and off-the-job embeddedness, moral leadership, and authoritarian leadership had small effect sizes. The research model was further extended with the moderating effect of environmental dynamism in predicting employee job performance. Results of moderating analysis confirmed that environmental dynamism negatively moderated the relationship between employee innovative work behavior and employee job performance. The predictive relevance of the research model was assessed with blindfolding procedure *Q*^2^ and confirmed the substantial predictive relevance to predict both endogenous variable innovative work behavior and employee job performance. These findings established that the research model had substantial power to predict employee innovative work behavior and job performance. A *post-hoc* analysis namely importance performance matrix analysis was employed to determine the importance and performance of the integrated research model. Findings revealed that constructs like benevolent leadership, moral leadership, innovative work behavior, and on-the-job embeddedness were the most influential factors to predict employee job performance. Therefore, this study concluded that human resource managers and policy makers should focus on benevolent leadership, moral leadership style, employee innovative work behavior, and on-the-job embeddedness in order to boost employee job performance.

### Limitations and Directions for Future Research Directions

This research has some limitations that impute future research directions. First, for empirical analysis, data were collected from small medium enterprises and we excluded large and well established organizations. Future researchers may include observations from multinational organizations in a dataset to broaden the research scope. The second limitation of this study is the respondent’s designation in the organization. The dataset included middle-level employees for required responses. Therefore, it is expected that paternalistic leadership style job embeddedness characteristics may vary for lower-level employees. Third, this study was cross sectional and investigated phenomenon at one point of time. Investigating this research model in a longitudinal context could reveal interesting findings. Finally, the research model was tested in the Saudi region and includes SME data only from the Saudi region, therefore replicating this research model in other regions may enhance the generalizability of the research model.

## Data Availability Statement

The original contributions presented in the study are included in the article/supplementary material, further inquiries can be directed to the corresponding author.

## Ethics Statement

The studies involving human participants were reviewed and approved by University of Jeddah. The patients/participants provided their written informed consent to participate in this study.

## Author Contributions

MY has acted as only author and led the writing and editorial process of this submission.

## Conflict of Interest

The author declares that the research was conducted in the absence of any commercial or financial relationships that could be construed as a potential conflict of interest.

## Publisher’s Note

All claims expressed in this article are solely those of the authors and do not necessarily represent those of their affiliated organizations, or those of the publisher, the editors and the reviewers. Any product that may be evaluated in this article, or claim that may be made by its manufacturer, is not guaranteed or endorsed by the publisher.

## Figures and Tables

**APPENDIX 1:**
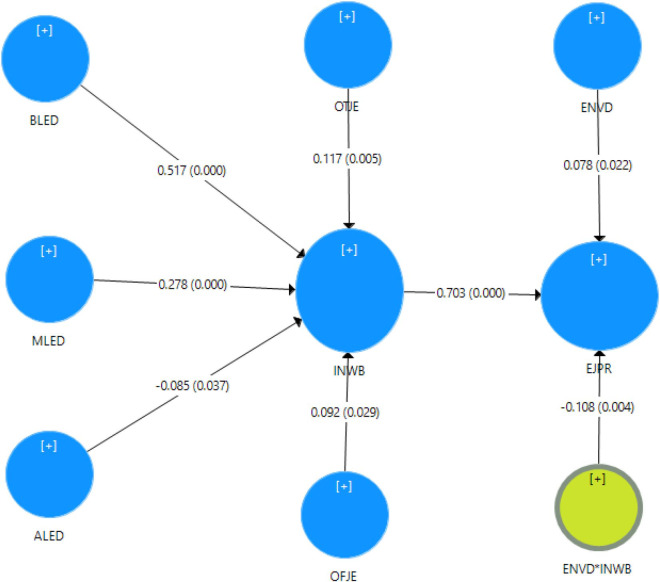
Path coefficient and significance level.
